# Factors associated with postpartum hemorrhage in selected Southern Oromia hospitals, Ethiopia, 2021: an unmatched case-control study

**DOI:** 10.3389/fgwh.2024.1332719

**Published:** 2024-03-14

**Authors:** Belda Negesa Beyene, Dube Jara Boneya, Shiferaw Gelchu Adola, Seboka Abebe Sori, Hirut Dinku Jiru, Nurye Sirage, Abdurazak Awol, Girma Tufa Melesse, Zelalem Jabessa Wayessa, Ahmedteyib Jemalo, Abebaw Kebede, Derese Eshetu, Yesuneh Dejene

**Affiliations:** ^1^ Department of Midwifery, Institute of Health, Bule Hora University, Bule Hora, Ethiopia; ^2^ Department of Public Health, College of Health Sciences, Debre Markos University, Debre Markos, Ethiopia; ^3^ Department of Nursing, Institute of Health, Bule Hora University, Bule Hora, Ethiopia; ^4^ Department of Midwifery, College of Medicine and Health Sciences, Wolkite University, Wolkite, Ethiopia; ^5^ Department of Midwifery, College of Health Sciences, Woldia University, Woldia, Ethiopia; ^6^ Department of Midwifery, College of Medicine and Health Sciences, Madda Walabu University, Bale Robe, Ethiopia; ^7^ Department of Midwifery, College of Medicine and Health Sciences, Wachemo University, Hossana, Ethiopia

**Keywords:** postpartum hemorrhage, maternal hemorrhage, determinants, Bule Hora, Ethiopia

## Abstract

**Background:**

Around one-fourth of maternal deaths worldwide are attributed to hemorrhage. One of the prevalent types of maternal hemorrhage is postpartum hemorrhage. In spite of this, there is very little data on postpartum hemorrhage. Thus, the intention of this study was to determine factors associated with postpartum hemorrhage among mothers who gave birth in the selected Southern Oromia hospitals in Ethiopia.

**Methods:**

An institutional-based, unmatched case-control study was employed on a sample of 333 (83 cases and 250 controls) mothers who gave birth from March 1–30, 2021. Standardized and pretested check-lists were used to retrieve data from patients' cards, delivery registration, and operation registration logbooks. Epi Data Version 3.1 was used to enter data, while SPSS Version 25 was used for analysis. Multi-variable logistic regressions were used to identify the determinants of postpartum haemorrhage with a 95% confidence interval and *p*-value less than 0.05.

**Results:**

Mothers who have no antenatal care follow-up (AOR = 1.94, 95% CI = 1.03, 3.64), had pre-partum anemia (AOR = 5.68, 95% CI = 3.13, 10.32), hypertensive disorder during pregnancy (AOR = 3.3, 95% CI = 1.13, 9.64), intrauterine fetal death (AOR = 4.21, 95% CI = 1.68, 10.58), and genital tract trauma during delivery (AOR = 3.23, 95% CI = 1.52, 6.89) were found as determinants of postpartum haemorrhage.

**Conclusion:**

The study showed that factors such as lack of antenatal care, pre–partum anemia, pregnancy–related hypertension, intrauterine fetal death and genital tract trauma during delivery were responsible for postpartum hemorrhage. The early introduction of antenatal care services for all mothers plays a crucial role in reducing postpartum hemorrhage.

## Introduction

Globally, maternal hemorrhage is the primary cause of preventable maternal death and morbidity (1–3). In Africa, maternal hemorrhage is one of the most common causes of maternal death, contributing from 26% to 45% (4–6).

Postpartum hemorrhage is one of the common types of maternal hemorrhage, which can be classified as primary or early postpartum hemorrhage (occurring within 24 h after childbirth) and secondary or late postpartum hemorrhage (after 24 h up to six weeks postpartum period) (7).

In Sub-Saharan Africa, postpartum hemorrhage is one cause of maternal death that is very difficult to manage due to the problem of implementing evidence-based practice since they are low-resource countries (8). In Ethiopia, postpartum hemorrhage accounts for 18.6% of maternal deaths (9).

Postpartum hemorrhage is defined as a blood loss of 500 millilitres or more for spontaneous vaginal delivery, or 1,000 millilitres for cesarean delivery or bleeding resulting in derangement of vital signs or a drop in greater than 10% of hematocrit from the baseline within 24 h after childbirth (10, 11).

Postpartum hemorrhage is mostly caused by uterine atony (failure of the uterus to contract), which accounts for about 70%–83.2%; trauma (laceration of the uterus, cervix, and vagina), which accounts for 10%–15%; and tissue (retained placenta and abnormal adherent placental attachment) (12, 13).

According to available evidence, maternal age ≥ 35 years old (14, 15), premature rupture of membrane (14), anemia (16, 17), previous caesarean delivery (18), multiple pregnancies (17), hypertension during pregnancy (19), prolonged labor (18), and not following antenatal care (20–22) are factors associated with postpartum hemorrhage.

More attention and investigation are still needed, according to contemporary researchers, more attention and investigation are still needed to fully understand the clinical features of primary postpartum hemorrhage (19, 23). There have been limited studies on primary postpartum hemorrhage in Ethiopia (21, 24–26).

The COVID-19 pandemic obstacles to maternal health include disrupting healthcare services, making healthcare providers scarce, preventing women from visiting hospitals out of fear of contracting an infection, and triggering contractions in the uterus to become irregular after delivery due to stress and pandemic anxiety (27).

According to the WHO's roadmap to combat postpartum hemorrhage between 2023 and 2030, key steps in eradicating preventable deaths from PPH include implementing the PPH research agenda, prioritizing research initiatives tackling implementation challenges, and improving coordination of innovations. To drive advancements and foster global collaboration among researchers, industry stakeholders, and innovators, research should be tailored to underserved populations, with active engagement of women and frontline healthcare providers, particularly midwives (11).

As a result, the major goal of this study was to determine factors associated with postpartum hemorrhage among mothers who gave birth at selected Southern Oromia hospitals in Ethiopia.

## Methods and materials

### Study design, period, and area

An institutional-based, unmatched case-control study was conducted from May 1–30, 2021, in the selected Southern Oromia hospitals in Ethiopia. It contains three zones that include Borena, West Guji, and East Guji Zone.

Borena, West Guji, and East Zone have contained 14 (8 woredas, 1 town, and 6 districts), 10 (9 woredas and 1 town), and 17 (8 woredas, 3 towns, and 6 districts), respectively. The capital city and their respective distances from Addis Ababa in the Borena, West Guji, and East Guji zones were Yebalo (575 km), Bule Hora (467 km), and Nagele (560 km), respectively. According to the 2013 population projection, the total population of Borena, West Guji, and East Guji Zones was 1,365,753, 1,422,767, and 1,303,732, respectively.

Southern Oromia has around 4 general hospitals (Adola, Nagele, Bule Hora, and Yabelo). Hospitals provide services like obstetrics, gynaecology, emergency, medical, surgical, laboratory, and radiology. Each of these hospitals has at least one gynaecologist, four integrated emergency surgeons, and more than fifteen midwives. The general hospital had a total delivery rate of at least 2,000 deliveries per year.

### Population

Source populations were all mothers who gave birth at selected Southern Oromia hospitals in the Southern Oromia region of Ethiopia.

Cases were mothers who gave birth in the selected hospitals, were diagnosed as PPH by the healthcare professionals during the study period, and were documented in the delivery logbook. Controls were mothers who delivered in the selected public hospitals but were not diagnosed with PPH by the healthcare professionals during the study period and were registered in the delivery logbook. In both groups, the maternal chart that had incomplete documentation was excluded.

### Sample size and sampling procedure

The sample size was calculated using Epi Info statistical software by considering the double population proportion formula. The proportion of exposure among controls was 88.8% (20). By considering the basic assumptions of 80% power, 95% CI, odd ratio 12.6, and *r* = 3, the calculated sample size was 76 cases and 227 controls. After assuming a 10% incomplete chart, the total sample size was 333 (83 cases and 250 controls).

First, three hospitals were purposefully selected out of nine hospitals, depending on their case flow. Then, three-year (1 September 2017 to 30 August 2020) data on women who gave birth at selected hospitals was extracted from the delivery registration log book. Then, lists of both cases and controls were identified from the registration logbook of the selected hospital. A proportional allocation was made for the calculated sample size among the three hospitals. A simple random sampling technique was used to select the sample of both groups from the identified list of cases and controls using computer-generated random numbers. Three controls were selected for one case from the list of both cases and controls.

### Data collection tool and procedure

Data were retrieved by three senior bachelor's degree midwives using a pretested and adapted check list from the previous literature (16, 28). The check list consists of socio-demographic, antepartum, and intrapartum factors. The principal investigator supervised the overall data collection process.

**Study Variables**
**Dependent variable**
  Postpartum hemorrhage
**Independent Variables**
  Socio-demographic-related factors: maternal age, residence

Antepartum-related factors: ANC follow-up, gravidity, parity, placenta previa, placenta abruption, number of fetuses, PROM, polyhydramnios, previous C/S delivery, history of abortion, history of stillbirth, Intrauterine fetal death, prenatal hemoglobin, hypertensive disorder, and magnesium sulphate.

Intrapartum-related factors: duration of active labour, obstructed labor, infection during labor, malpresentation, augmentation or induction of labor, mode of delivery, episiotomy, genital tract trauma, retained tissue, uterine atony, the weight of the newborn, and gestational age at delivery.

### Operational definition

Cases: mothers who gave birth, documented in the logbook, and diagnosed with PPH by healthcare providers.

Controls: mothers who gave birth documented in the logbook but not diagnosed with PPH by healthcare providers.

Incomplete documents: those charts that lack essential documents like a history sheet, delivery summary, or both are used to assess the outcome variables.

### Data quality assurance

Two days of training were given to data collectors and supervisors to familiarize them with the prepared checklist, the data retrieval procedures, and the objective of the study. A pretest was done on the 5% sample (4 cases and 12 controls) at Kercha Hospital to check the consistency. After pretesting, variables like obstructed labour were added, and antepartum haemorrhage was among the modified variables. Close supervision was carried out by the principal investigator and supervisor during data retrieval to check the completeness, clarity, consistency, and accuracy of the retrieved data.

### Data processing and analysis

The data were checked for accuracy, consistency, and completeness. Then, it was entered into Epi Data version 3.1 and exported to SPSS version 25 for data cleaning and analysis. Descriptive statistics such as median, frequency, and percentages were used to describe the study subject's characteristics about relevant variables.

Logistic regression was fitted to assess the association between dependent and independent variables. Bi-variable analysis was employed to select candidate variables for the multi-variable model. Those variables that show an association with primary postpartum hemorrhage at a *p*-value ≤ 0.25 were transferred into the multi-variable logistic regression model. The crude and adjusted odds ratios, together with their 95% confidence intervals, were used to identify the strength of the association.

An assumption of logistic regression was checked before the last multi-variable analysis using the variance inflation factor and was not detected. The model's goodness of fit was checked using the Hosmer-Lemeshow test, and its value became insignificant (0.35). A *p*-value of less than 0.05 was used to describe the statistical significance of the findings in this study. The final result was presented using text, tables, and graphs based on the types of data.

## Results

### Socio-demographic characteristics of mothers

There were 250 controls and 83 cases (333 mother's charts) in this study. The median age of the mothers was 26 (IQR 9) and 25.0 (IQR 8) years among cases and controls, respectively. About 51 (61.5%) of cases and 88 (35.2%) of controls were living in rural areas (Table 1).

**Table 1:** Socio-demographic characteristics of mothers for the study of PPH in the selected Southern Oromia Hospitals, Ethiopia, 2021.

Variables	Category	Cases *N* (%)	Controls *N* (%)	Total *N* (%)
Hospital	Adola General Hospital	34 (41)	76 (30.4)	110 (33)
	Bule Hora General Hospital	13 (15.6)	91 (36.4)	104 (31.2)
	Yebalo General Hospital	36 (43.4)	83 (33.2)	119 (35.8)
Residence	Urban	32 (38.6)	162 (64.8)	194 (58.3)
	Rural	51 (61.4)	88 (35.2)	139 (41.7)
Age	15–19 years	9 (10.8)	34 (13.6)	43 (12.9)
	20–24 years	21 (25.3)	86 (34.4)	107 (32.1)
	25–29 years	30 (36.1)	80 (32)	110 (33)
	30–34 years	13 (15.7)	28 (11.2)	41 (12.3)
	>35 years	10(12)	22(8.8)	32(9.6)

### Antepartum characteristics of the mothers

A large percentage of cases (94%) and controls (95.2%) have never delivered C/S before. In terms of placental anomalies, 13 mothers (10 cases and 3 controls) have an abruption placenta, while 21 mothers (13 cases and 8 controls) have a placenta previa. Premature rupture of the membrane is not present in 228 (91.4%) of the controls and the majority of cases (90.4%). Polyhydramnios appears to be absent in 100% of the controls and in the majority of cases (98.2%). Around 8 cases (9.6%) and 14 controls (5.6%) of pregnant mothers have hypertensive disorders; of them, 14 mothers (6 cases and 8 controls) received magnesium sulphate (Table 2).

**Table 2:** Antepartum characteristics of mothers for the study of PPH in the selected Southern Oromia Hospitals, Ethiopia, 2021.

Variables	Category	Cases *N* (%)	Controls *N* (%)	Total *N* (%)
Gravidity	Primigravida	21 (25.3)	80 (32)	101 (30.3)
	Multigravida	38 (45.8)	131 (52.4)	169 (50.7)
	Grand multigravida	24 (28.9)	39 (15.6)	63 (19)
Parity	Nulliparous	23 (27.7)	88 (35.2)	111 (33.3)
	Primipara	16 (19.3)	55 (22)	71 (21.3)
	Multipara	24 (28.9)	84 (33.6)	108 (32.4)
	Grandmultipara	20 (24.0)	23 (9.2)	43 (13)
History of abortion	Yes	6 (7.2)	31 (12.4)	37 (11.1)
	No	77 (92.8)	219 (87.6)	332 (88.9)
History of stillbirth	Yes	3 (3.6)	8 (3.2)	11 (3.3)
	No	80 (96.4)	242 (96.8)	322 (96.7)
ANC follow-up	Yes	44 (53)	193 (77.2)	237 (71.2)
	No	39 (47)	57 (22.8)	96 (28.8)
Previous	Yes	5 (6)	12 (4.8)	17 (5.1)
C/S delivery	No	78 (94)	238 (95.2)	316 (9.5)
Gestation age	<37 wk	42 (50.6)	107 (42.8)	149 (44.7)
	37–42 wk	41 (49.4)	143 (57.2)	184 (55.3)
Prenatal Hemoglobin	<11 gr/dl	59 (71)	63 (25.2)	122 (36.6)
	≥11 gr/dl	24(29)	187(74.8)	211(63.4)

ANC, antenatal care; C/S, caesarean delivery.

### Intrapartum characteristics of the mother

About thirty-eight (12 cases and 26 controls) mothers have used oxytocin during labor. Eleven (5 cases and 6 controls) mothers have obstructed labour during their labour. Three (3.6%) of cases and eleven (4.4%) of controls have malpresentation reported. Singleton delivery status was reported by the majority of the cases (92.7%) and controls (96.4%). In the current study, 9 (10.8%) cases and 41 (16.4%) controls had an episiotomy during birth, whereas 19 (22.9%) cases and 30 (12%) controls also had genital tract trauma other than an episiotomy (Table 3).

**Table 3:** Intrapartum characteristics of respondents in the selected Southern Oromia hospitals, Ethiopia, 2021.

Variables	Category	Cases *N* (%)	Controls *N* (%)	Total *N* (%)
Infection during labor	Yes	1 (1.2)	9 (3.6)	10 (3)
	No	82 (98.2)	241 (96.4)	323 (97)
Duration of labor (From 4 cm-delivery)	<8 h	65 (78.3)	225 (90)	290 (87)
	≥8 h	18 (21.7)	25 (10)	75 (13)
Baby birth weight	<2,500 g	17 (20.5)	26 (10.4)	43 (13)
	2,500–3,999 g	49 (59)	195 (78)	244 (73.2)
	≥ 4,000 g	17 (20.5)	29 (11.6)	46 (13.8)
Mode of delivery	SVD	52 (62.6)	187 (74.8)	239 (71.7)
	ID	7 (8.4)	25 (10)	32 (9.6)
	C/S	24 (29)	38 (15.2)	62 (18.6)
Intrauterine fetal death	Yes	20 (24.1)	11 (4.4)	31 (9.3)
	No	63(75.9)	239(95.6)	302(90.7)

SVD, spontaneous vaginal delivery; ID, instrumental delivery; C/S, caesarean delivery.

### Determinant factors of postpartum hemorrhage

In multi-variable logistic regression, women who had no ANC follow-up during pregnancy, women who had prenatal anemia, hypertensive disorder during pregnancy, IUFD, and genital tract trauma during delivery were found to be determinants of PPH.

Women who had no ANC follow-up during pregnancy were almost two times higher than those who had ANC follow-up [AOR = 1.94, 95% CI (1.03, 3.64)]. Women with the hypertensive disorder during pregnancy had 3.3 times higher odds of developing PPH compared to those who did not have the hypertensive disorder [AOR = 3.3, 95% CI (1.13, 9.64)].

Women who had prenatal anaemia (Hgb < 11 g/dl) had 5.7 times higher odds of developing primary PPH compared to those who did not have prenatal anaemia (Hgb > 11 g/dl) during pregnancy [AOR = 5.68, 95% CI (3.13, 10.32)]. Women who had intrauterine foetal death were 4.2 times more likely to develop PPH as compared to those who had no intrauterine foetal death [AOR = 4.21, 95% CI (1.68, 10.58)].

The odds of developing PPH among women who had genital tract trauma during delivery were 3.2 times more likely than those who had no genital tract trauma during delivery [AOR = 3.23, 95% CI (1.52, 6.89)] (Table 4).

**Table 4:** Bivariable and multivariable logistic regression analysis of PPH in the selected Southern Oromia hospitals, Ethiopia, 2021.

Variables	Category	Cases	Controls	COR (95% CI)	AOR (95% CI)	*P*-value
		(*N* = 83)	(*N* = 250)			
ANC	Yes	44 (53)	193 (77.2)	1	1	
	No	39 (47)	5 7 (22.8)	3.00 (1.78, 5.06)	1.94 (1.03,3.64)*	0.039
IUFD	Yes	20 (24.1)	11 (4.4)	6.89 (3.14,15.14)	4.21 (1.68,10.58)*	0.002
	No	63 (75.9)	239 (95.6)	1	1	
HTN	Yes	8 (9.6)	14 (5.6)	1.79 (0.73,4.45)	3.30 (1.13,9.64)*	0.029
	No	75 (90.4)	236 (94.4)	1	1	
Placenta previa	Yes	13 (15.7)	8 (3.2)	5.62 (2.24,14.01)*	2.80 (0.97–8.09)	0.057
	No	70 (84.3)	242 (96.8)	1	1	
Duration of labour	<8 h	65 (78.3)	225 (90)	1	1	
	≥8 h	18 (21.7)	25 (10)	2.49 (1.28, 4.85)	1.83 (0.81,4.13)	0.144
Prenatal hemoglobin	<11 gr/dl	59 (71)	63 (25.2)	7.29 (4.19,12.69)	5.68 (3.13,10.32)*	<0.001
	≥11 gr/dl	24 (29)	187 (74.8)	1	1	
Trauma	Yes	19 (22.9)	30 (12)	2.17 (1.15,4.12)*	3.23 (1.52,6.89)	0.002
	No	64(77.1)	220(88)	1	1	

* Statistically significant at *p*-value < 0.05, 1.

ANC, antenatal care; AOR, adjusted odd ratio; COR, crude odd ratio, CI, confidence interval; IUFD, intrauterine foetal death; HTN, hypertension.

## Discussion

The study findings highlight significant factors contributing to postpartum hemorrhage, including the absence of antenatal care, pre-partum anemia, pregnancy-related hypertension, intrauterine fetal death, and genital tract trauma during delivery. Taking proactive measures such as implementing universal antenatal care and thorough assessments of diverse risk factors could be crucial in reducing the occurrence of postpartum hemorrhage.

In this study, women who had antenatal care follow-up were less likely to experience PPH, compared to those mothers who had not antenatal care follow-up. A study undertaken in Ethiopia at Dessie Referral, Debra Tabor General, Hiwot Fana Specialized University Hospital, and Southern Tigray (20–22, 26) supports this conclusion. The ultrasound scan, which is part of prenatal care and can identify early PPH risk markers such as placenta previa, abruption, and uterine anomalies, can be missed by the mother if there is no ANC follow-up (29). This finding was inconsistent with a study done at Douala General Hospital, Cameroon (30). This might be due to the low proportion of women who had ANC follow-up in the current study, and having ANC follow-up might get preventive measures for PPH from health care providers.

According to the current study, one of the determinant factors for postpartum hemorrhage during pregnancy was hypertensive disorder. This result is consistent with the studies done in Norway (16), Alsager teaching hospitals (31), and South Ethiopia (17). The possible justification might be due to the fact that pregnant women with hypertensive disorders may experience PPH due to abnormalities in the uterus, delayed placental delivery, abnormalities in the placenta, and alterations in vascular resistance (32, 33). The other reason might be that magnesium sulphate was administered to women as a treatment for hypertensive disorder, and this drug has a vasodilation effect that leads to uterine atony after delivery (34). However, findings from the study conducted at Hiwot Fana specialized hospital in Eastern Ethiopia (22) showed that no association exists between complications during pregnancy like hypertension and postpartum hemorrhage. This could be due to more service accessibility in the referral hospital in the previous study and the difference in study design.

Compared to women without prenatal anemia, those with prenatal anemia had a 5.7-fold increased risk of postpartum hemorrhage. This result is in line with a study conducted in Norway (16), China (35), Udaipur (36), Egypt (37), and South Ethiopia (17). This may be due to the fact that mothers who have prenatal anemia encounter uterine hypoxia as a result of hemoglobin reduction, which results in an inadequate uterine contraction and uterine atony (38).

This study found that women who suffered intrauterine foetal death had a 4.2 times higher likelihood of postpartum hemorrhage in comparison to those who did not. This finding is supported by a study at Chattogram General Hospital, Bangladesh (39), at a tertiary care centre in greater Noida, India (40), and at N'Djamena Mother and Child University Hospital, West Africa (41). This may be explained by the fact that IUFD may lead to PPH by missing part of the placental, prolonged duration of labour that leads to inadequate uterine contraction, and the use of oxytocin as a medical intervention.

The study also showed that genital tract trauma other than episiotomy was also an important determinant factor for postpartum haemorrhage. This finding agrees with the study conducted in Japan (19), Afghanistan (28), and Egypt (37). This could be due to deep laceration of the vagina, which might sometimes involve arteries and arterioles, and cervical laceration may occur due to rapid dilation of the cervix during labor and delivery, which is difficult for individual suturing and leads to primary PPH. This finding was inconsistent with the study done at Debra Tabor, Ethiopia (21). This could be due to study design differences and the fact that a high proportion of women who gave birth had genital tract trauma in this study. The absence of genital tract trauma may help to avoid blood loss as a result of trauma.

Variables like the abnormal third stage of labor, the lack of active management of labor, and the absence of labor monitoring by partograph were not assessed (26).

### Strengths and limitations of the study

This study's strength is that it employed simple random sampling procedures to reduce selection bias in the process of choosing both cases and controls from the same source group of people. Pre-testing of the checklist was conducted, and any necessary adjustments were made. However, a limitation of this study is the reliance on secondary data from records, which resulted in the omission of some variables.

## Conclusion

This study demonstrated that factors associated with postpartum hemorrhage included genital tract trauma, lack of antenatal care follow-up, being anemic during pregnancy, having hypertensive disorder, and experiencing intrauterine fetal mortality.

### Recommendations

The government should prioritize accessibility of healthcare facilities and professional development of healthcare providers, specifically skills in the management of postpartum haemorrhage. This focus is vital, as enhanced use of ANC services can mitigate the risk of postpartum hemorrhage and its related complications. The zonal office of the Maternal and Child Health Bureau should take the initiative to raise awareness about antenatal care services for all women. Healthcare professionals should deliver comprehensive ANC services by counseling pregnant women on improved nutritional intake during pregnancy and providing iron and folate supplements.

Healthcare professionals are advised to diagnose and treat hypertensive disorders during pregnancy, IUFD, and prenatal anaemia as soon as possible. Once more, it is advised that obstetric care providers closely monitor and treat any genital tract trauma sustained during delivery.

## Data Availability

The raw data supporting the conclusions of this article will be made available by the authors, without undue reservation.
